# Isolation and FTIR-ATR and ^1^H NMR Characterization of Alginates from the Main Alginophyte Species of the Atlantic Coast of Morocco

**DOI:** 10.3390/molecules25184335

**Published:** 2020-09-22

**Authors:** Zahira Belattmania, Soukaina Kaidi, Samir El Atouani, Chaimaa Katif, Fouad Bentiss, Charafeddine Jama, Abdeltif Reani, Brahim Sabour, Vitor Vasconcelos

**Affiliations:** 1 R.U. Phycology, Blue Biodiversity & Biotechnology—P3B, Laboratory of Plant Biotechnology, Ecology and Ecosystem Valorization, Faculty of Sciences, Chouaïb Doukkali University, P.O. Box 20, El Jadida M-24000, Morocco; belattmania.z@ucd.ac.ma (Z.B.); souk.kaidi@gmail.com (S.K.); elatouanisamir@gmail.com (S.E.A.); chaimaakatif25@gmail.com (C.K.); abreani@yahoo.fr (A.R.); sabour.b@ucd.ac.ma (B.S.); 2 Laboratory of Catalysis and Corrosion of Materials, Faculty of Sciences, Chouaïb Doukkali University, P.O. Box 20, El Jadida M-24000, Morocco; fbentiss@gmail.com; 3 University of Lille, CNRS, INRAE, Centrale Lille, UMR 8207, UMET—Unité Matériaux et Transformations, F-59000 Lille, France; Charafeddine.Jama@ensc-lille.fr; 4 CIIMAR, Interdisciplinary Centre of Marine and Environmental Research, University of Porto, Terminal de Cruzeiros do Porto de Leixões, Av. General Norton de Matos, s/n, 4450-208 Matosinhos, Portugal; 5 Departament of Biology, Faculty of Sciences, University of Porto, Rua do Campo Alegre, 4169-007 Porto, Portugal

**Keywords:** brown seaweeds, alginates, spectroscopic characterization, Morocco

## Abstract

Alginates are widely used as gelling agents in textile print pastes, medical industries, impression material in dentistry, and anticoagulant material in toothpaste. In the present study, the content and spectroscopic characterization (^1^H NMR and FT-IR) of the sodium alginates were investigated in the eight brown seaweeds *Sargassum muticum*, *Fucus vesiculosus* f. *volubilis*, *Carpodesmia tamariscifolia*, *Bifurcaria bifurcata*, *Laminaria ochroleuca*, *Cystoseira humilis*, *Saccorhiza polyschides*, and *Fucus guiryi* harvested from the NW Atlantic coast of Morocco. The results proved that the most studied algae depicted alginate yields higher than 18% dry weight. The FT-IR analysis showed that the spectra of the extracted alginates exhibited significant similarities to the commercial alginate from Sigma-Aldrich. The ^1^H NMR spectroscopy indicated that the extracted alginates have a high content of β-d-mannuronic (M) than α-l-guluronic acid (G) with M/G ratio values ranging from 1.04 to 4.41. The homopolymeric fractions F_MM_ are remarkably high compared to the F_GG_ and heteropolymeric fractions (F_GM_ = F_MG_) especially for *F. guiryi*, *C humilis*, *C. tamariscifolia*, *L. ochroleuca*, and *S. polyschides*. Nevertheless, the heteropolymeric fractions (F_GM_/F_MG_) are quite abundant in the alginates of *S. muticum*, *F. vesiculosus* f. *volubilis*, and *B. bifurcata* accounting for more than 52% of the polymer diads. Based on these results, the investigated algal species (except *Fucus guiryi* and *Bifurcaria bifurcata*) could be regarded as potential sources of alginates for industrial uses.

## 1. Introduction

Macroalgae are documented mainly as a source of critical chemical components with applications in human food, phycocolloids, cosmeceuticals, and pharmaceutical industries, as well as the agricultural sector for incorporation into animal feeds and plant stimulants [1]. Three types of phycocolloids are commonly involved in the seaweed chemical industry such as alginates, carrageenans, and agars. Alginate is the common name given to a family of linear phycocolloid β-d-mannuronic (1,4-linked) and α-l-guluronic acids arranged in a non-regular, blockwise order along the chain [2]. This polysaccharide is found in the matrix and the cell wall of the brown algae. It plays a vital role in cell binding and makes available some mechanical properties to the algae, e.g., flexibility [3]. The viscosity of alginate hydrocolloids depends on the average molecular weight, whereas the gelling properties are affected by the amounts and distribution of the three types of blocks (The homopolymeric fractions of β-d-mannuronic (F_M_) and α-l-guluronic (F_G_) acids, and the heteropolymeric fractions (F_MG_) [1,3]). In general, alginates with a low M/G ratio and a large proportion of guluronic blocks form a robust and rigid gel. Those with a low number of guluronic blocks and a high M/G ratio produce soft and elastic gels [4]. Alginates are widely used as stabilizers in various food industries, textile print pastes, gelling agents in medical industries, impression material in dentistry, and anticoagulant material in toothpaste [1,5]. Several brown algal species are cultivated to produce the alginate required for the industry such as *Ascophyllum nodosum*, *Durvillaea antarctica*, *Laminaria hyperborea*, *Saccharina latissima*, *Ecklonia maxima*, *Macrocystis pyrifera*, *Lessonia nigrescens*, and *Lessonia trabeculata* [6,7].

The Atlantic coast of Moroccan is a favorable habitat for diverse algal species and constitutes a reserve of species of considerable economic, social, and ecologic potentials. Nevertheless, the phycocolloid industry is limited to the exploitation of *Gelidium corneum* (Gelidiales, Rhodophyceae), which is commercially harvested for agar extraction. Indeed, several other red and brown algae are abundant on this coast, but the data on the composition and physicochemical properties of their polysaccharides are rare. In particular, there is no alginate industry in Morocco, and almost no information is available on potential alginophytes species and the quantity and quality of alginates. In this context, the present study explores the extraction yield, as well as the spectroscopic characterization (^1^H NMR and FT-IR) of the sodium alginates from eight brown algal species (*Sargassum muticum*, *Fucus vesiculosus* f. *volubilis*, *Carpodesmia tamariscifolia*, *Bifurcaria bifurcata*, *Laminaria ochroleuca*, *Cystoseira humilis*, *Saccorhiza polyschides*, and *Fucus guiryi*) harvested from the Atlantic coast of Morocco.

## 2. Results and Discussion

### 2.1. Alginates’ Yield

The alginate yields of the eight brown algal species collected from the Northwestern Atlantic coast of Morocco ranged from 2.7% to 27.5% dw (Table 1). The highest percent of alginates was obtained from *Laminaria ochroleuca*, and the minimum alginate content was found in *Bifurcaria bifurcata*. The alginates’ yield recorded in *S. muticum*, *C. tamariscifolia*, *C. humilis* and *F. vesiculosus* exceeded 18% dw. These contents are within the range of those reported from some well-known worldwide alginophytes such as *Saccharina japonica* (20–26% dw [8,9]), *Ascophyllum nodosum* (24% [10]), and *Saccharina longicruris* (20% dw [10]). However, these values are still lower in comparison to the special alginate contents of *Durvillaea antarctica* (from 37 to 52% [11]) and *Ecklonia cava* from (35 to 38% dw [9]). The alginate content observed in *F. guiryi* (13.6% dw) is still higher than that previously reported from others Fucales species (10–12% dw [12,13]). The kelp species *L. ochroleuca* and *S. polyschides* contained 27.5% and 25% alginates, respectively (Table 1). Similar values have been reported for *Laminaria digitata* (22–34% dw [8,14]), *Laminaria hyperborea* (21–33% dw [14,15]), and *Macrocystis pyrifera* (29–38% dw [11]).

### 2.2. FT-IR Spectroscopy Analysis

**Figure 1:**
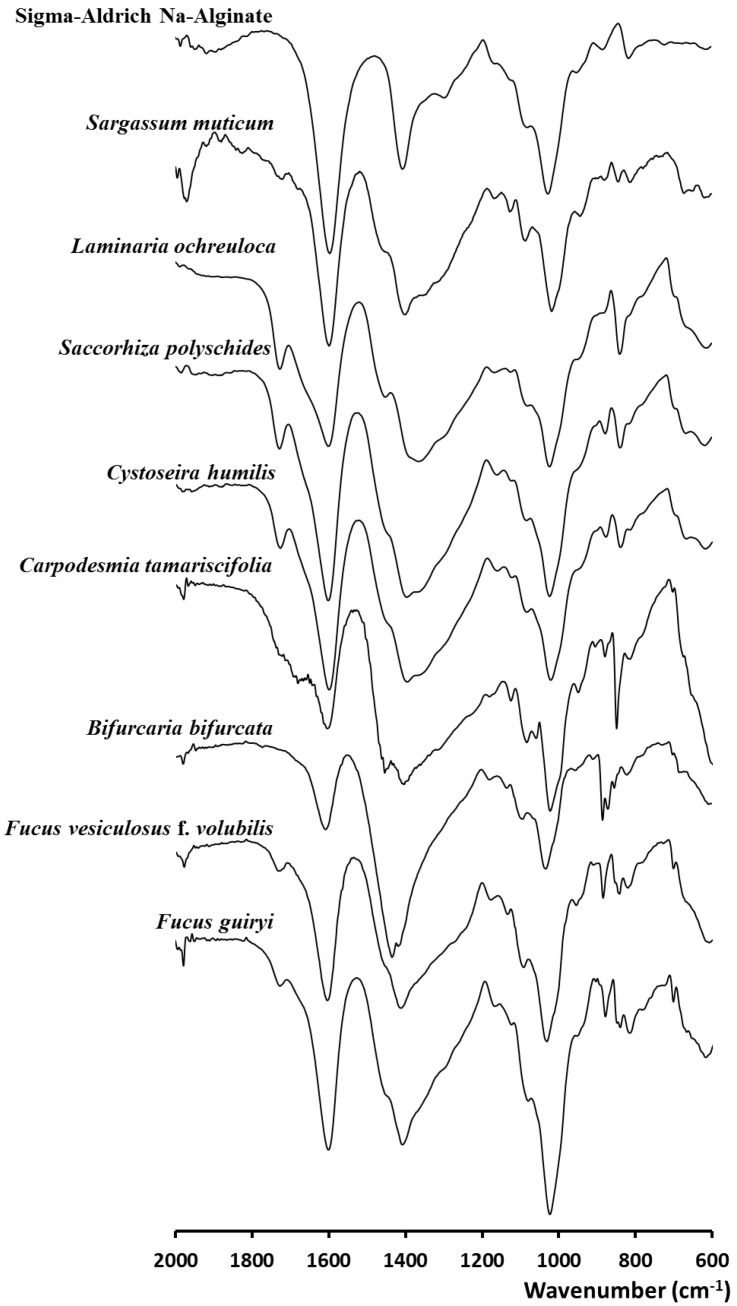
FT-IR spectra of sodium alginates extracted from the investigated brown seaweeds and the sodium alginate standard (Sigma-Aldrich, Gillingham, UK).

The infrared spectra in the range 2000 to 600 cm^−1^ of alginates extracted from the investigated brown algae with reference to commercial sodium alginate (Sigma-Aldrich, Gillingham, UK) are given in Figure 1. The spectra of extracted alginates are very similar to those of the commercial standard showing similar positions of the characteristic bands. Therefore, sodium alginate is the main polysaccharide found in the tested brown seaweeds, despite the small-signal appearing between 1710 cm^−1^ and 1730 cm^−1^, particularly in the spectra of *Fucus guiryi*, *S. muticum*, *S. polyschides*, *L. ochroleuca*, and *F. vesiculosus*, corresponding to the carbonyl group as the carboxylic acid ester form (C=O). The abroad bands at 1600–1610 cm^−1^ were suggested as the O-C-O carboxylate asymmetric stretching [23,24]. The bands located at 1400–1428 cm^−1^ were assigned to C-OH deformation vibration with the involvement of the symmetric stretching vibration of O-C-O [13,25]. According to previous reports, the bands at 1025–1030 cm^−1^ can be attributed to the C-O group [18]. The anomeric region (950 to 750 cm^−1^) is the most discussed in carbohydrates [23,26]. Indeed, the C-O stretching vibration of uronic acid residues is generally linked to the bands centered around 930–950 cm^−1^, and those recorded between 871 and 883 cm^−1^ were attributed to the C1-H deformation vibration of β-mannuronic acid residues [27]. The signals around 815–833 cm^−1^ were attributed to mannuronic acid residues [27].

### 2.3. ^1^H NMR Spectroscopy Analysis

**Figure 2:**
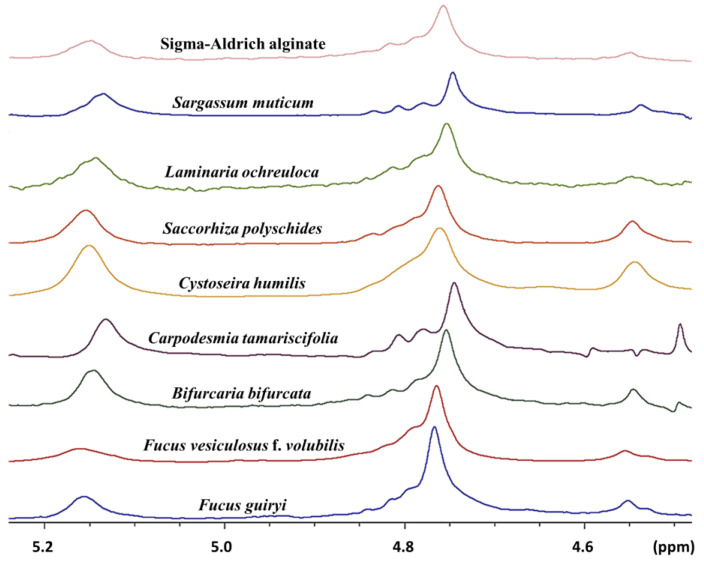
^1^H NMR spectra of the studied sodium alginates using D_2_O as a solvent.

^1^H NMR spectroscopy is a consistent method for the complete characterization of the composition and the block structure of alginate [11,12]. The phycocolloids extracted from the studied brown algae revealed typical 400 MHz-^1^H NMR spectra (Figure 2) similar to the commercial sodium alginate with three key signals attributed to the anomeric hydrogen of guluronic acid (G) at 5.1–5.2 ppm (pic I), the anomeric hydrogens of mannuronic acid (M1), and the H-5 of alternating blocks (GM-5) overlapping at 4.7–4.9 ppm (pic II), and the H-5 of guluronic acid residues in the homopolymeric G blocks, at 4.5–4.6 ppm (pic III) (Figure S1 in the Supplementary Material).

The M/G ratio, the molar fractions of the monads (F_G_, F_M_), and the diad (F_GG_, F_MM_, F_MG_, F_GM_) sequences were calculated from the area of ^1^H NMR signals (Figure 2) employing the formula given by Grasdalen et al. [28]:

F_G_ = A_I_/(A_II_ + A_III_)

F_M_ = 1 − F_G_

F_GG_ = A_III_/(A_II_ + A_III_)

F_GM_ = F_MG_ = F_G_ − F_GG_

F_MM_ = F_M_ − F_MG_

M/G = (1 − F_G_)/F_G_

The M/G ratios of sodium alginates in all tested brown algae were greater than one (Table 2), revealing a dominance of mannuronic acid over guluronic acid, in particular in *L. ochroleuca* and *F. guiryi*, showing the M/G ratios of 2.3 and 4.4, respectively. Such dominance has been reported for alginates in *Durvillaea antarctica* [11] and for the analyzed commercial alginate (Sigma-Aldrich, Gillingham, UK). According to Murillo-Álvarez and Hernández-Carmona [29], alginates with a low M/G ratio would provide strong, brittle gels, making them suitable for cell encapsulation for biomedical or environmental applications, whereas alginates with a high M/G ratio would give more elastic gels, desirable for food, cosmetic, or pharmaceutical products. The M/G ratio is not the only factor that controls the alginate gelling properties. It has been reported that the percentages of homopolymeric block structures (F_MM_, F_GG_) and alternating blocks (F_MG_, F_GM_) influence the physical properties of alginates [19]. Based on the ^1^H NMR results (Table 2), the homopolymeric regions (F_MM_) are remarkably high compared to the guluronic blocks (F_GG_) and heteropolymeric fractions (F_GM_ = F_MG_) for *F. guiryi*, *C humilis*, *C. tamariscifolia*, *L. ochroleuca*, and *S. polyschides* and the tested commercial alginate. Similar compositions have previously been found for *Durvillaea antarctica*, *Saccharina japonica*, *Laminaria digitata*, and *Macrocystis pyrifera* [8,11]. The heteropolymeric fractions (F_GM_/F_MG_) are quite abundant in *S. muticum*, *F. vesiculosus*, and *B. bifurcata*, accounting for more than 52% of the polymer diads.

The description of the alginate sequence could be completed via the parameter *η* = F_MG_/(F_M_ × F_G_), which evaluates and reveals the distribution of sequences. Indeed, *η* values <1 correspond to the abundance of homopolymeric blocks MM and GG, *η* = 1 for completely random cases and 1 < *η* < 2 for alternate-like cases MG and GM [28].

The *η* values exceeded one for the alginates extracted from *S. muticum*, *B. bifurcata*, and *F. vesiculosus* (Table 2), proving the dominance of the heteropolymeric fractions MG and GM. The *η* values were less than one for *C. tamariscifolia*, *C. humilis*, and *F. guiryi*, reflecting the abundance of homopolymeric fractions MM and GG. Nonetheless, *L. ochroleuca* and *S. polyschides* showed *η* values equal to one with a codominance of homopolymeric and heteropolymeric blocks.

## 3. Materials and Methods

The studied brown algal species (Figure S2 in the Supplementary Material) were sampled from the El Jadida coastline (33°14′43.7″ N 8°32′35.2″ W), except *F. vesiculosus* f. *volubilis*, which was harvested from Oualidia lagoon (32°44′52.7″ N 9°01′26.8″ W). In the laboratory, the samples were washed with running tap water and then with deionized water to remove debris sticking to their surface. Algal biomasses were dried at 60 °C until constant weight.

The alginates’ extraction was carried out according to the slightly modified procedure of Calumpong et al. [30]. Dried biomasses of the investigated algal species was covered with 2% formaldehyde at room temperature for 24 h, washed with water, and then added to 0.2 M HCl and left for another 24 h. Subsequently, the algal biomasses were rinsed with distilled water and flooded in 2% sodium carbonate for 24 h. The soluble part was collected by filtration through three layers of cheesecloth, and the filtrate was collected by centrifugation. The extract was discarded, and the procedure was repeated for the solid residue. The whole filtrates were precipitated by three volumes of ethanol. The sodium alginate recovered was washed with acetone and placed in an oven at 50 °C. The yield of alginate is expressed as a percentage of the initial dry weight of seaweed (% dw).

FTIR spectral measurements of the dried sodium alginate samples (50 °C for 3h) were performed using a Thermo Scientific Nicolet Impact 400D FT-IR Spectrometer (Nicolet Instrument Co., Madison, USA). The spectra were scanned between 4000 and 600 cm^−1^ in attenuated total reflectance (ATR) mode. A total of 32 scans were averaged for each sample at a 4 cm^−1^ resolution, and subsequently, the IR spectra were processed using the OMNIC software (Nicolet, Madison, USA).

The ^1^H NMR spectra of the sodium alginate solutions in D_2_O were recorded on the spectrometer AV II 400 MHz, 9.4T (Proton Larmor frequency of 400.33 MHz, Bruker Corporation, Billerica, MA, USA), using A 5 mm Triple Resonance Broadband Inverse probe (Bruker Corporation, Billerica, MA, USA), at 343 K. The spectra of 16 K in size of Free Induction Decay were recorded with a sweep width of 4800 Hz. Presaturation was applied during the relaxation delay and mixing time. The raw data were apodized in one dimension with 0.5 for line broadening prior to Fourier transformation. The number of scans was 32 transients. A commercial sodium alginate (CAS No. 9005-38-3, Lot MKBQ4519V, Sigma-Aldrich, Gillingham, UK) was used as the standard.

## 4. Conclusions

The present study investigates the yield and spectroscopic (FT-IR and ^1^H NMR) characterization of alginates extracted from eight brown seaweeds from the Moroccan Atlantic coastline. The alginates contents were higher than 18% dw for most of the algae except *F. guiryi* and *B. bifurcata*. FT-IR spectroscopy analysis showed an interesting similarity between the alginate spectra of the studied algae and that of the commercial alginate (Sigma-Aldrich, Gillingham, UK). ^1^H NMR spectroscopy revealed that the extracted alginates are richer in mannuronic acid than guluronic acid (M/G ratio >1), thus providing elastic hydrogels. The quasi-dominance of mannuronic (F_MM_) and guluronic (F_GG_) homopolymers over heteropolymeric fractions (F_GM_ and F_MG_) was detected in *F. guiryi*, *C. humilis*, and *C. tamariscifolia*. However, the heteropolymeric fractions dominated the diads of the alginates extracted from *S. muticum*, *B. bifurcata*, and *F. vesiculosus*. The noteworthy yield associated with the physical and chemical proprieties of the extracted alginates makes these species potential alginophytes for commercial purposes. Nonetheless, future research must place emphasis on the seasonal variation of yield and the composition of the extracted phycocolloids.

## Figures and Tables

**Table 1:** Alginate contents of the investigated algal species compared to various brown seaweeds.

Algal Species	Alginates’ Yield (% dw)	References
*Sargassum muticum*	25.6	This study
*Laminaria ochroleuca*	27.5	
*Saccorhiza polyschides*	25.0	
*Cystoseira humilis*	19.1	
*Carpodesmia tamariscifolia*	17.22	
*Fucus vesiculosus* f. *volubilis*	18.3	
*Fucus guiryi*	13.6	
*Ascophyllum nodosum*	24	[10]
*Durvillaea antarctica*	37–52	[11]
*Ecklonia maxima*	35	[8]
*Laminaria japonica*	20–26	[8]
*Laminaria digitata*	22–34	[8]
*Laminaria hyperborea*	21–33	[15]
*Lessonia nigrescens*	34–41	[16]
*Macrocystis pyrifera*	29–38	[11]
*Saccharina longicruris*	20	[10]
*Sargassum asperifolium*	12	[12]
*Sargassum filipendula*	17	[17]
*Sargassum fluitans*	21	[18]
*Sargassum vulgare*	17	[19]
*Sargassum muticum*	18	[20]
*Sargassum oligocystum*	19	[18]
*Sargassum thunbergii*	13	[20]
*Sargassum polycystum*	17–28	[20]
*Sargassum turbinarioides*	10	[13]
*Fucus vesiculosus*	16.2	[10]
*Fucus serratus*	20–29	[21]
*Fucus ceranoides*	21–29	[22]

**Table 2:** Composition data of alginates extracted from studied seaweeds compared to other brown seaweed species.

Species	M/G	F_M_	F_G_	F_MM_	F_GG_	F_GM_	F_MG_	*η*	References
*Sargassum muticum*	1.04	0.51	0.49	0.17	0.15	0.34	0.34	1.35	
*Laminaria ochroleuca*	2.52	0.72	0.28	0.50	0.06	0.22	0.22	1.09	
*Fucus guiryi*	4.41	0.82	0.18	0.78	0.15	0.04	0.04	0.27	
*Cystoseira humilis*	1.46	0.59	0.41	0.40	0.21	0.20	0.20	0.83	
*Carpodesmia tamariscifolia*	1.31	0.57	0.43	0.42	0.28	0.15	0.15	0.61	This study
*Saccorhiza polyschides*	1.73	0.63	0.37	0.38	0.11	0.25	0.25	1.09	
*Bifurcaria bifurcata*	1.88	0.65	0.35	0.33	0.02	0.32	0.32	1.43	
*Fucus vesiculosus* f. *volubilis*	1.84	0.65	0.35	0.39	0.09	0.26	0.26	1.13	
Sigma-Aldrich Na-Alginate	3.42	0.77	0.23	0.68	0.14	0.09	0.09	0.51	
*Ascophyllum nodosum*	0.85	0.46	0.54	0.28	0.36	0.18	0.18	0.72	[10]
*Durvillaea antarctica*	4.00	0.8	0.2	0.64	0.04	0.16	0.16	1.00	[11]
*Ecklonia maxima*	1.22	0.55	0.45	0.32	0.22	0.23	0.23	0.93	[8]
*Laminaria japonica*	1.86	0.65	0.35	0.48	0.18	0.17	0.17	0.75	[8]
*Laminaria digitata*	1.44	0.59	0.41	0.43	0.25	0.16	0.16	0.66	[8]
*Laminaria hyperborea*	0.82	0.45	0.55	0.28	0.38	0.17	0.17	0.69	[15]
*Lessonia nigrescens*	1.44	0.59	0.41	0.4	0.22	0.19	0.19	0.79	[12]
*Macrocystis pyrifera*	1.7	0.63	0.37	0.42	0.16	0.21	0.21	0.90	[11]
*Saccharina longicruris*	0.69	0.41	0.59	0.07	0.25	0.34	0.34	1.41	[10]
*Sargassum asperifolium*	0.69	0.41	0.59	0.3	0.48	0.22	0.22	0.91	[12]
*Sargassum filipendula*	0.78	0.44	0.56	0.33	0.45	0.11	0.11	0.45	[17]
*Sargassum muticum*	0.31	0.24	0.76	0.07	0.59	0.17	0.17	0.93	[20]
*Sargassum oligocystum*	0.62	0.38	0.62	0.31	0.55	0.14	0.14	0.59	[18]
*Sargassum thunbergii*	0.53	0.34	0.66	0.17	0.48	0.34	0.34	1.52	[20]
*Sargassum polycystum*	0.21	0.18	0.82	0.12	0.77	0.1	0.1	0.68	[20]
*Sargassum vulgare*	1.27	0.56	0.44	0.02	0.55	0.43	0.43	1.75	[19]
*Fucus vesiculosus*	1.44	0.59	0.41	0.39	0.22	0.19	0.19	0.78	[10]

## Supplementary Materials

The following are available online. **Figure S1:** The anomeric region in the 400 MHz—^1^H NMR spectrum of the Sigma-Aldrich sodium alginate using D2O as solvent. **Figure S2:** Morphological traits of the studied macroalgae collected on the Northwestern Atlantic coast of Morocco.

## References

[1] Stengel B.D., Connan S., Stengel B.D., Connan S. (2015). Marine Algae: A Source of Biomass for Biotechnological Applications. Natural Products From Marine Algae: Methods and Protocols.

[2] Andrade L., Salgado L.T., Farina M., Pereira M.S., Mourão P.A., Filho G.M.A. (2004). Ultrastructure of acidic polysaccharides from the cell walls of brown algae. J. Struct. Boil..

[3] Kloareg B., Quatrano R. (1988). Structure of the cell walls of marine algae and ecophysiological function of the matrix polysaccharides. Oceanogr. Mar. Biol..

[4] Bourgougnon N., Stiger-Pouvreau V. (2011). Chemodiversity and Bioactivity within Red and Brown Macroalgae Along the French coasts, Metropole and Overseas Departements and Territories. Handbook of Marine Macroalgae.

[5] Hanzhi L., Song Q., Peng J. (2011). Biotechnology of Seaweeds: Facing the Coming Decade. Handbook of Marine Macroalgae.

[6] Helgerud T., Gaserød O., Fjæreide T., Andersen P.O., Larsen C.K., Imeson A. (2009). Alginates. Food Stabilizers, Thickeners and Gelling Agents.

[7] Rioux L.-E., Turgeon S.L. (2015). Seaweed carbohydrates. Seaweed Sustainability.

[8] Smidsrød O., Draget K.I. (1996). Alginates: Chemistry and physical properties. Carbohydr. Eur..

[9] Pérez R., Pérez R. (1997). Les extraits des végétaux marins: Les phycocolloïdes. Ces algues qui nous entourent: Conception actuelle, rôle dans la biosphère, utilisations, culture.

[10] Rioux L.-E., Turgeon S.L., Beaulieu M. (2007). Characterization of polysaccharides extracted from brown seaweeds. Carbohydr. Polym..

[11] Panikkar R., Brasch D.J. (1996). Composition and block structure of alginates from New Zealand brown seaweeds. Carbohydr. Res..

[12] Larsen B., Salem D.M., Sallam M.A., Mishrikey M.M., Beltagy A.I. (2003). Characterization of the alginates from algae harvested at the Egyptian Red Sea coast. Carbohydr. Res..

[13] Fenoradosoa T.A., Ali G., Delattre C., Laroche C., Petit E., Wadouachi A., Michaud P. (2009). Extraction and characterization of an alginate from the brown seaweed Sargassum turbinarioides Grunow. Environ. Boil. Fishes.

[14] Schiener P., Black K.D., Stanley M.S., Green D.H. (2014). The seasonal variation in the chemical composition of the kelp species Laminaria digitata, Laminaria hyperborea, Saccharina latissima and Alaria esculenta. Environ. Boil. Fishes.

[15] Martinsen A., Skjåk-BraeK G., Smidsrød O. (1989). Alginate as immobilization material: I. Correlation between chemical and physical properties of alginate gel beads. Biotechnol. Bioeng..

[16] Donati I., Paoletti S. (2009). Material Properties of Alginates.

[17] Bertagnolli C., Espindola A.P.D., Kleinübing S.J., Tasic L., Da Silva M.G.C. (2014). Sargassum filipendula alginate from Brazil: Seasonal influence and characteristics. Carbohydr. Polym..

[18] Davis T.A., Ramirez M., Mucci A., Larsen B. (2004). Extraction, isolation and cadmium binding of alginate from Sargassum spp.. Environ. Boil. Fishes.

[19] Torres M.R., Sousa A.P., Filho E.A.S., Melo D.F., Feitosa J.P., De Paula R.C., Lima M.G. (2007). Extraction and physicochemical characterization of Sargassum vulgare alginate from Brazil. Carbohydr. Res..

[20] Davis T.A., Llanes F., Volesky B., Mucci A. (2003). Metal Selectivity of *Sargassum* spp. and Their Alginates in Relation to Their α-l-Guluronic Acid Content and Conformation. Environ. Sci. Technol..

[21] Haug A. (1964). Composition and Properties of Alginates.

[22] Hoppe H.A., Schmid O.J. (1962). Meeresalgen als moderne Industrie produkte. Bot. Mar..

[23] Mathlouthi M., Koenig J.L. (1987). Vibrational Spectra of Carbohydrates. Adv. Carbohydr. Chem. Biochem..

[24] Bi F., Mahmood S.J., Arman M., Taj N., Iqbal S. (2007). Physicochemical characterization and ionic studies of sodium alginate fromSargassum terrarium(brown algae). Phys. Chem. Liq..

[25] Papageorgiou S.K., Kouvelos E.P., Favvas E.P., Sapalidis A., Romanos G.E., Katsaros F. (2010). Metal-carboxylate interactions in metal-alginate complexes studied with FTIR spectroscopy. Carbohydr. Res..

[26] Leal D., Matsuhiro B., Rossi M., Caruso F. (2008). FT-IR spectra of alginic acid block fractions in three species of brown seaweeds. Carbohydr. Res..

[27] Gómez-Ordóñez E., Jiménez-Escrig A., Rupérez P. (2010). Dietary fibre and physicochemical properties of several edible seaweeds from the northwestern Spanish coast. Food Res. Int..

[28] Grasdalen H., Larsen B., Smidsrød O. (1979). A p.m.r. study of the composition and sequence of uronate residues in alginates. Carbohydr. Res..

[29] Murillo-Alvarez J.I., Hernández-Carmona G. (2007). Monomer composition and sequence of sodium alginate extracted at pilot plant scale from three commercially important seaweeds from Mexico. Environ. Boil. Fishes.

[30] Calumpong H.P., Maypa A.P., Magbanua M. (1999). Population and alginate yield and quality assessment of four Sargassum species in Negros Island, central Philippines. Hydrobiologia.

