# Supplementary material for: Isolation and FTIR-ATR and 1H NMR Characterization of Alginates from the Main Alginophyte Species of the Atlantic Coast of Morocco
Source: Molecules. 2020 Sep 22;25(18):4335. doi: 10.3390/molecules25184335 (PMC7570942; doi:10.3390/molecules25184335)
Supplement: Supplementary file 1 [file molecules-25-04335-s001.pdf]

molecules

MDPI

Supplementary Material

Isolation and FTIR-ATR and ^{1}H NMR Characterization of Alginates from the Main Alginophyte Species of the Atlantic Coast of Morocco

Zahira Belattmania [1], Soukaina Kaidi [1], Samir El Atouani [1], Chaimaa Katif [1], Fouad Bentiss [2,3], Charafeddine Jama [3], Abdeltif Reani [1], Brahim Sabour [1] and Vitor Vasconcelos [4,5,*]

[1] R.U. Phycology, Blue Biodiversity & Biotechnology – P3B, Laboratory of Plant Biotechnology, Ecology and Ecosystem Valorization, Faculty of Sciences, Chouaïb Doukkali University, PO Box 20, M-24000 El Jadida; belattmania.z@ucdac.ma (Z.B.); souk.kaidi@gmail.com (S.K.); elatouanisamir@gmail.com (S.E.); chaimaakatif25@gmail.com (C.K.); abreani@yahoo.fr (A.R.); sabour.b@ucd.ac.ma (B.S.)

[2] Laboratory of Catalysis and Corrosion of Materials, Faculty of Sciences, Chouaïb Doukkali University, PO Box 20, M-24000 El Jadida, Morocco; fbentiss@gmail.com (F.B.)

[3] University of Lille, CNRS, INRAE, Centrale Lille, UMR 8207, UMET – Unité Matériaux et Transformations, F-59000 Lille, France; charafeddine.jama@ensc-lille.fr (C.J.)

[4] CIIMAR, Interdisciplinary Centre of Marine and Environmental Research, University of Porto, Terminal de Cruzeiros do Porto de Leixões, Av. General Norton de Matos, s/n, 4450-208 Matosinhos, Portugal

[5] Departament of Biology, Faculty of Sciences, University of Porto, Rua do Campo Alegre, 4169-007 Porto, Portugal

* Correspondence: vmvascon@fc.up.pt; Tel.: 351-223401817 (V.V.)

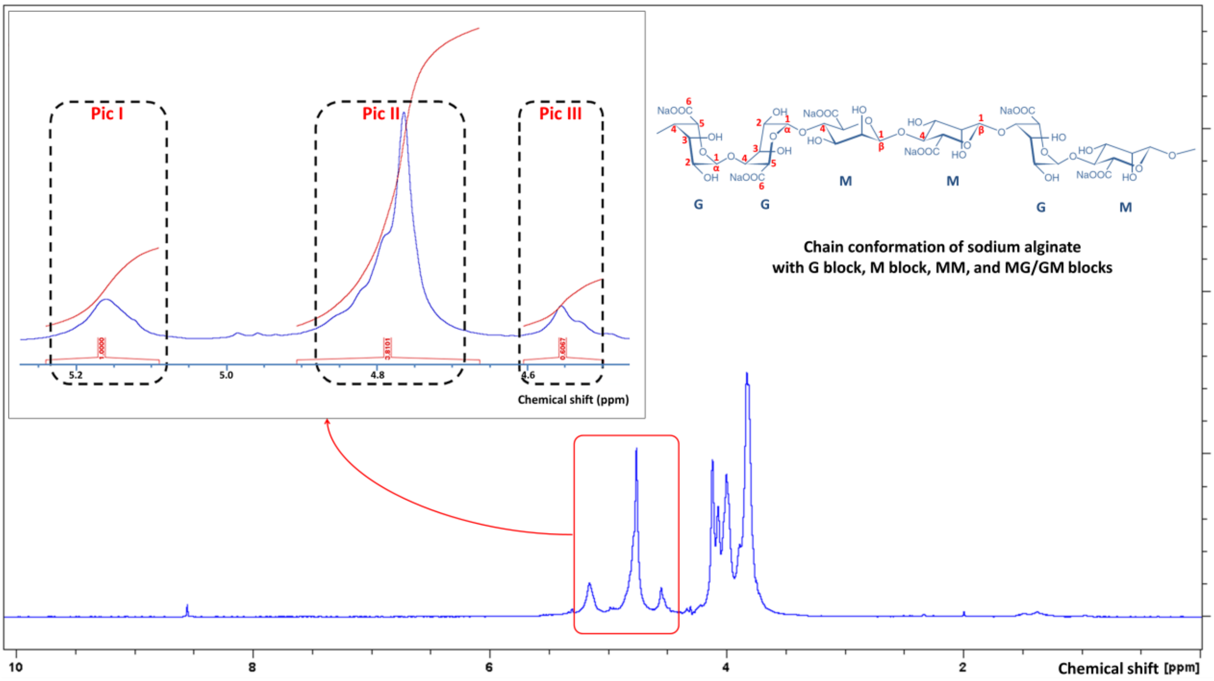

Figure S1. The anomeric region in the 400 MHz - ^{1}H NMR spectrum of the Sigma-Aldrich sodium alginate using D_2O as solvent.

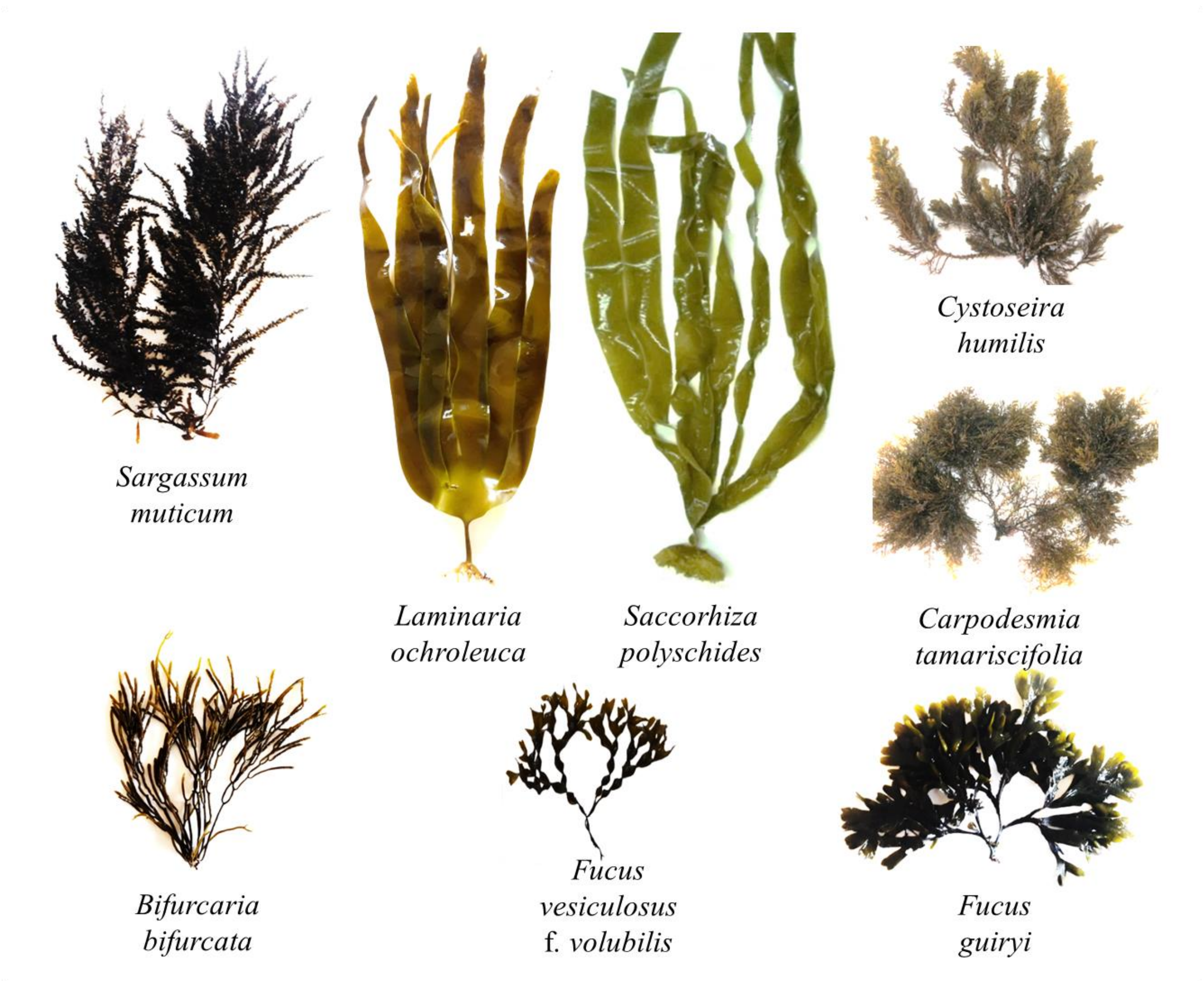

Figure 2S. Morphological traits of the studied macroalgae collected on the Northwestern Atlantic coast of Morocco.
